# Chewing difficulty, swallowing problems, xerostomia and cause‐specific mortality among older adults: A 7‐year follow‐up JAGES cohort study

**DOI:** 10.1111/ggi.70150

**Published:** 2025-08-25

**Authors:** Taro Kusama, Yudai Tamada, Sakura Kiuchi, Masashige Saito, Toshiyuki Ojima, Jun Aida, Katsunori Kondo, Ken Osaka, Kenji Takeuchi

**Affiliations:** ^1^ Division of Statistics and Data Science, Liaison Center for Innovative Dentistry Tohoku University Graduate School of Dentistry Miyagi Japan; ^2^ Department of International and Community Oral Health Tohoku University Graduate School of Dentistry Miyagi Japan; ^3^ Department of Preventive Medicine Nagoya University Graduate School of Medicine Aichi Japan; ^4^ Frontier Research Institute for Interdisciplinary Sciences Tohoku University Miyagi Japan; ^5^ Faculty of Social Welfare Nihon Fukushi University Aichi Japan; ^6^ Department of Community Health and Preventive Medicine Hamamatsu University School of Medicine Shizuoka Japan; ^7^ Department of Dental Public Health, Graduate School of Medical and Dental Sciences Institute of Science Tokyo Tokyo Japan; ^8^ Center for Preventive Medical Sciences Chiba University Chiba Japan; ^9^ Research Department Institute for Health Economics and Policy Tokyo Japan

**Keywords:** dysphagia, frailty, longitudinal study, mastication, oral health

## Abstract

**Aim:**

Although various poor oral health conditions have been reported to be associated with an increased all‐cause mortality risk, a comprehensive investigation of the causes of death is limited. This study aimed to investigate the association between poor oral function and mortality due to various causes in older adults.

**Methods:**

This 7‐year follow‐up cohort study used data from the Japan Gerontological Evaluation Study. The participants were independent older adults aged ≥65 years at baseline. The outcome and exposure variables were 10 types of cause‐specific mortality and poor oral functioning (chewing difficulty, swallowing problems and xerostomia). Cause‐specific hazard ratios (HRs) and 95% confidence intervals (95% CIs) were estimated using the Cox proportional hazards model.

**Results:**

Among the 44 083 participants (mean age 73.7 years [SD 6.0], men 46.8%), the all‐cause mortality was 20.76 per 1000 person‐years. After considering all covariates, chewing difficulty was significantly associated with high mortality risk due to neoplasms (HR 1.18, 95% CI 1.07–1.30) and circulatory diseases (HR 1.15, 95% CI 1.01–1.31), swallowing problems was significantly associated with high mortality risk due to nervous system diseases (HR 2.84, 95% CI 1.80–4.47) and respiratory diseases (HR 1.42, 95% CI 1.20–1.70), and xerostomia was significantly associated with high mortality risk due to nervous system diseases (HR 1.87, 95% CI 1.20–2.92), circulatory diseases (HR 1.20, 95% CI 1.06–1.37) and respiratory diseases (HR 1.35, 95% CI 1.16–1.58), respectively.

**Conclusions:**

The present results suggest that poor oral functioning, including chewing difficulty, swallowing problems and xerostomia, could amplify the risk of mortality due to specific diseases. Dental and medical professionals should pay more attention to older adults with poor oral function to prevent subsequent critical health issues. **Geriatr Gerontol Int 2025; 25: 1332–1340**.

## Graphical abstract

- Poor oral functioning could amplify the risk of mortality due to specific diseases in older adults.
- Dental and medical professionals should pay more attention to older adults with poor oral function to prevent subsequent critical health issues.

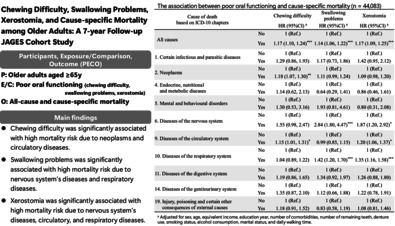

## Introduction

Poor oral health is one of the most prevalent health issues worldwide.^1^ Dental diseases, including dental caries, periodontal disease and tooth loss, are highly prevalent among older adults because of their irreversible nature.^2^, ^3^, ^4^ Additionally, recent studies have emphasized that the deterioration of oral function due to aging, such as chewing difficulty, dysphagia and dry mouth, is a critical health problem among older adults.^5^ With global aging, the importance of oral health problems will increase from a public health perspective.

Previous studies have shown an association between poor oral health, including dental diseases and poor oral functioning, and an increased risk of all‐cause mortality.^6^, ^7^, ^8^, ^9^ Additionally, several studies have reported an association between tooth loss and cause‐specific mortality due to cardiovascular diseases, cancer and respiratory diseases.^10^, ^11^, ^12^, ^13^, ^14^ These findings contribute to the elucidation of the underlying mechanisms of oral and systemic health. For poor oral functioning, previous studies have reported that poor mastication, dysphagia and dry mouth are associated with high all‐cause mortality risks.^7^, ^8^, ^9^ However, although some previous studies investigated the association between tooth loss and cause‐specific mortality,^15^ few studies have investigated the cause of mortality due to poor oral function, and investigation of the underlying mechanism is lacking. Furthermore, limiting the analysis to selected cause‐specific mortalities as outcomes cannot eliminate the potential bias introduced by the non‐reporting of non‐significant results.^16^ Therefore, a comprehensive approach using various cause‐specific mortalities as outcomes is essential to fully elucidate the association between oral health and cause‐specific mortality.

Oral function is affected by both dental diseases and physiological changes due to aging.^5^ Poor oral functioning is considered multifaceted and not completely explained by the influence of dental diseases, such as tooth loss.^5^ Therefore, the cause of the increased mortality risk due to poor oral functioning might differ from that of tooth loss. The decline in oral function leads to various health problems from both medical and social aspects, including malnutrition, social inactivity, disabilities and mortality.^17^ Additionally, the health burden of poor oral functioning would increase owing to a global aging society. Therefore, it is also essential to elucidate the impact of poor oral function on cause‐specific mortality, a key indicator of public health, to maintain favorable health in later life.

We hypothesized that poor oral functioning could affect mortality risk irrespective of the dental condition and that the risk would differ by cause. This exploratory study aimed to investigate the association between each component of poor oral functioning, including chewing difficulty, swallowing problems, xerostomia and mortality due to various causes, in older adults.

## Methods

### Study design and participants

This cohort study was based on self‐reported questionnaires and administrative databases with a 7‐year follow‐up period. We used data from the Japan Gerontological Evaluation Study (JAGES), a large cohort study of adults aged ≥65 years in Japan,^18^ who did not receive public long‐term care insurance. Survey data from 11 municipalities were included. Baseline questionnaire surveys were carried out in 2010, and mortality was followed from baseline based on the administrative databases of municipalities. At baseline, questionnaires were sent to the participants by mail, after which they were retrieved if they consented to participate. We excluded participants whose activities of daily living were not independent at baseline.

### Outcome variables

We used all‐cause and cause‐specific mortality as outcome variables. Data on the incidence of death and its causes were retrieved from the government administrative database based on Vital Statistics. The causes of death were classified based on the International Classification of Diseases and Related Health Problems ver. 10 (ICD‐10). We used the ICD‐10 chapter‐level classification. In the present study, the incidence of death in some chapter‐level classifications was quite low among the analyzed population; therefore, we did not use these classifications to avoid bias due to sparse outcomes. The cause of death included the following 10 chapter‐level classes as outcome variables: “chap. 1: Certain infectious and parasitic diseases,” “chap. 2: Neoplasms,” “chap. 4: Endocrine, nutritional, and metabolic diseases,” “chap. 5: Mental and behavioral disorders,” “chap. 6: Diseases of the nervous system,” “chap. 9: Diseases of the circulatory system,” “chap. 10: Diseases of the respiratory system. chap. 11: Diseases of the digestive system,” “chap. 14: Diseases of the genitourinary system” and “chap. 19: Injury, poisoning and other consequences of external causes.” We also included all‐cause mortality as an outcome variable. For additional analysis, we utilized more detailed data on the causes of death, categorized using a simplified classification system developed by the Japanese government. In the current study dataset, the number of deaths attributable to each disease was small. Consequently, these data were considered only supplementary information owing to the challenge involved in achieving sufficiently narrow confidence intervals.

### Exposure variables

We used self‐reported poor oral function, including chewing difficulty, swallowing problems and xerostomia, as an exposure variable. The participants were asked to answer “Yes” or “No” to “Do you have any difficulties eating tough foods compared with 6 months ago?” for chewing difficulty, “Have you choked on your tea or soup recently?” for swallowing problems, and “Do you often experience having a dry mouth?” for xerostomia. Those who answered “Yes” to each question were classified as having poor oral function. These questions were included in a validated questionnaire for screening frailty in older Japanese adults (Kihon Checklist).^19^ Furthermore, these three variables were also included in the Oral Frailty 5‐item Checklist, and there is a consensus statement that these questions reflect the oral function.^17^ The Oral Frailty 5‐item Checklist consists of fewer remaining teeth, chewing difficulty, swallowing problems, xerostomia and speaking difficulty. Although we were unable to include speaking difficulty, the components of oral function are considered to be reasonably represented by the three variables used in the present study, and the number of remaining teeth was also adjusted in the analysis. A previous cohort study showed that each question regarding poor oral functioning on the Kihon Checklist was significantly associated with the risk of functional disabilities.^20^ Therefore, we selected these three variables of oral function, including chewing difficulty, swallowing problems and xerostomia, as the exposures. In this study, we focused on each variable as an exposure for a more straightforward interpretation of the results and potential underlying mechanisms. Although the validity of each question of poor oral function that was used in the present study was not confirmed previously, previous studies have reported the validity of similar questions for subjective chewing ability, subjective swallowing problems and xerostomia, and they reflected clinical measurements.^21^, ^22^, ^23^ In addition, a previous study reported that individuals who answered that they had chewing difficulty in the questions asked in the present study also had worsened objective chewing ability.^24^

### Covariates

Based on previous studies and clinical knowledge, we included covariates at baseline as possible confounders.^7^, ^8^, ^9^, ^25^, ^26^ They included sex (male/female), age (65–69/70–74/75–79/80–84/≥85 years), equivalent income (<2.00/2.00–3.99/≥4.00 million JPY), education (<9/10–12/≥13 years), number of comorbidities (0/1/2/≥3), number of remaining teeth (≤19/≥20), denture use (Yes/No), smoking status (never/past/current), alcohol consumption (never/past/current), marital status (with/without a spouse) and daily walking time (<30/30–59/≥60 min/day) as physical activity.

### Statistical analysis

We fitted the Cox proportional hazards model, and estimated hazard ratios (HRs) and 95% confidence intervals (CIs). In the model that used cause‐specific mortality as an outcome, we treated death due to other causes as censoring, and estimated cause‐specific HRs.^27^ Although we excluded the causes of death with few incidences from the investigated outcomes, the incidence of several cause‐specific deaths remained low. Therefore, we used inverse probability weighting methods to adjust for covariates and avoid bias due to sparse outcomes.^28^ In the inverse probability weighting method, we estimated the propensity score for each exposure variable based on a logistic regression model, including all covariates. Subsequently, we calculated the average treatment effect weight and considered the weight in each model. We also analyzed all‐cause mortality.

To reduce selection bias, we carried out multiple imputations.^29^ We created five imputed datasets using multivariate imputation by chained equations. The estimates from each imputed dataset were combined based on Rubin's rule. For sensitivity analysis, we also analyzed the complete records. To identify the strength of the unmeasured covariates that affected the estimates, we calculated *E*‐values. The *E*‐value represents the minimum strength of the association required for the unmeasured confounder to have both exposure and outcome, conditional on the measured covariates explaining the association.^30^ The significance level was uniformly set at alpha = 0.05. The present study was based on individual hypothesis for each exposure and outcome variable, and corrections for multiple testing were not conducted.^31^ Stata/MP (version 17.0; StataCorp, College Station TX, USA) was used to carry out the statistical analyses.

## Results

**Figure 1:**
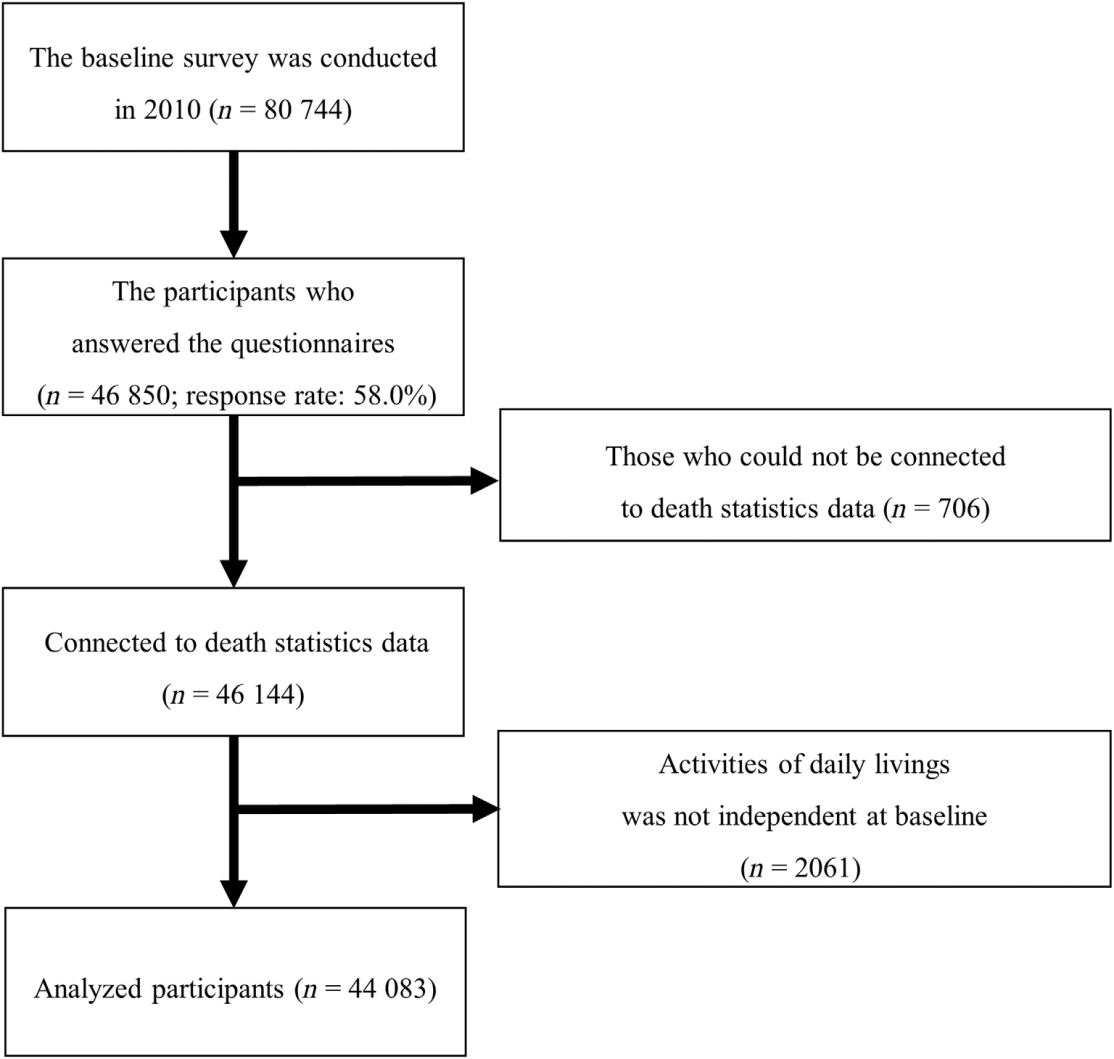
Flowchart of the participants’ inclusion.

Figure 1 shows a flowchart of participant inclusion. Finally, 44 083 participants were included in the analyzed population (mean age 73.7 years (SD 6.0), men 46.8%). Baseline characteristics before and after multiple imputations are presented in Table 1. The median follow‐up period was 2485 days (IQR 2218–2697 days). The overall mortality rate due to all‐cause death was 20.76 per 1000 person‐years.

**Table 1:** The characteristics distribution of the analyzed participants before and after multiple imputations (*n* = 44 083)

Characteristics		Original dataset		After MI
		*n*	%	%
Chewing difficulty	No	30 507	69.2	72.9
	Yes	11 229	25.5	27.1
	Missing	2347	5.3	‐
Swallowing problems	No	35 002	79.4	84.1
	Yes	6541	14.8	15.9
	Missing	2540	5.8	‐
Xerostomia	No	32 337	73.4	78.9
	Yes	8550	19.4	21.1
	Missing	3196	7.2	‐
Sex	Male	20 634	46.8	46.8
	Female	23 449	53.2	53.2
Age (years)	65–69	13 064	29.6	29.6
	70–74	13 178	29.9	29.9
	75–79	9839	22.3	22.3
	80–84	5519	12.5	12.5
	≥85	2483	5.6	5.6
Equivalent income (million JPY)	<2.00	18 039	40.9	50.2
	2.00–3.99	14 449	32.8	38.9
	≥4.00	4082	9.3	10.9
	Missing	7513	17.0	‐
Education (years)	≤9	19 571	44.4	45.6
	10–12	15 408	35.0	35.7
	≥13	8070	18.3	18.7
	Missing	1034	2.3	‐
No. comorbidities	0	10 084	22.9	25.0
	1	12 403	28.1	30.8
	2	8713	19.8	21.9
	≥3	8836	20.0	22.3
	Missing	4047	9.2	‐
No. remaining teeth	≥20	15 148	34.4	35.5
	≤19	27 417	62.2	64.5
	Missing	1518	3.4	‐
Denture use	No	18 110	41.1	46.0
	Yes	21 287	48.3	54.0
	Missing	4686	10.6	‐
Smoking status	Never	23 549	53.4	60.0
	Past	11 806	26.8	29.0
	Current	4516	10.2	11.0
	Missing	4212	9.6	‐
Alcohol consumption	Never	25 001	56.7	60.3
	Past	1446	3.3	3.5
	Current	15 083	34.2	36.2
	Missing	2553	5.8	‐
Marital status	With a spouse	31 320	71.1	72.5
	Without a spouse	11 815	26.8	27.5
	Missing	948	2.1	‐
Walking time (min/day)	≥60	13 019	29.5	31.3
	30–59	14 686	33.3	35.5
	<30	13 733	31.2	33.2
	Missing	2645	6.0	‐

Abbreviation: MI, multiple imputation.

Tables 2, 3, 4 show the all‐cause and cause‐specific mortality rates for each exposure, and the results of the Cox regression model. The mortality rate was high among patients with poor oral functioning, irrespective of the cause of death. After weighting by average treatment effect weight, the distributions of baseline characteristics between the exposed and unexposed groups were almost the same (Supplementary Tables S1–S3). Tables 2, 3, 4 present the regression model results using the inverse probability weighting method. Those with poor oral functioning were at a high risk of all‐cause mortality (for chewing difficulty, HR 1.17, 95% CI 1.10–1.24; for swallowing problems, HR 1.14, 95% CI 1.06–1.22; for xerostomia, HR 1.17, 95% CI 1.09–1.25). For each cause‐specific mortality, those with chewing difficulty were at a high risk of death due to neoplasms (HR 1.18, 95% CI 1.07–1.30) and circulatory diseases (HR 1.15, 95% CI 1.01–1.31). Those with swallowing problems were at a high risk of death due to nervous system diseases (HR 2.84, 95% CI 1.80–4.47]) and respiratory diseases (HR 1.42, 95% CI 1.20–1.70). Those with xerostomia were at a high risk of death due to nervous system's diseases (HR 1.87, 95% CI 1.20–2.92), circulatory diseases (HR 1.20, 95% CI 1.06–1.37) and respiratory diseases (HR 1.35, 95% CI 1.16–1.58). The estimated *E*‐values are presented in Supplementary Table S4. Sensitivity analysis of the complete records showed similar results (Supplementary Table S5).

**Table 2:** Association between chewing difficulty and cause‐specific mortality (*n* = 44 083)

Cause of death based on ICD‐10 chapters	Chewing difficulty	Incidence rate per 1000 PYs	Crude model	ATE‐IPW model^a^
			HR (95% CI)	HR (95% CI)
All causes	No	17.94	1 (Ref.)	1 (Ref.)
	Yes	28.60	1.60 (1.52, 1.70)***	1.17 (1.10, 1.24)***
1. Certain infectious and parasitic diseases	No	0.38	1 (Ref.)	1 (Ref.)
	Yes	0.69	1.82 (1.27, 2.62)***	1.29 (0.86, 1.93)
2. Neoplasms	No	7.69	1 (Ref.)	1 (Ref.)
	Yes	11.35	1.48 (1.36, 1.62)***	1.18 (1.07, 1.30)**
4. Endocrine, nutritional and metabolic diseases	No	0.19	1 (Ref.)	1 (Ref.)
	Yes	0.29	1.54 (0.85, 2.79)	1.14 (0.62, 2.13)
5. Mental and behavioral disorders	No	0.09	1 (Ref.)	1 (Ref.)
	Yes	0.14	1.58 (0.70, 3.57)	1.30 (0.53, 3.16)
6. Diseases of the nervous system	No	0.27	1 (Ref.)	1 (Ref.)
	Yes	0.53	1.97 (1.27, 3.06)**	1.55 (0.98, 2.47)
9. Diseases of the circulatory system	No	4.14	1 (Ref.)	1 (Ref.)
	Yes	6.68	1.62 (1.45, 1.82)***	1.15 (1.01, 1.31)*
10. Diseases of the respiratory system	No	2.49	1 (Ref.)	1 (Ref.)
	Yes	4.03	1.63 (1.41, 1.89)***	1.04 (0.89, 1.22)
11. Diseases of the digestive system	No	0.51	1 (Ref.)	1 (Ref.)
	Yes	0.95	1.88 (1.38, 2.55)***	1.19 (0.86, 1.65)
14. Diseases of the genitourinary system	No	0.31	1 (Ref.)	1 (Ref.)
	Yes	0.59	1.92 (1.28, 2.88)**	1.35 (0.87, 2.10)
19. Injury, poisoning and certain other consequences of external causes	No	0.85	1 (Ref.)	1 (Ref.)
	Yes	1.64	1.70 (1.33, 2.16)***	1.18 (0.91, 1.52)

^a^ Inverse probability weighting was conducted by using average treatment weight calculated by propensity score of each exposure variable. Each propensity score was estimated by all covariates, including sex, age, equivalent income, education years, number of comorbidities, number of remaining teeth, denture use, smoking status, alcohol consumption, marital status and daily walking time.

Abbreviations: 95% CI, 95% confidence interval; ATE, average treatment effect; HR, hazard ratio; ICD‐10, International Classification of Diseases and Related Health Problems ver. 10; IPW, inverse probability weighted; PYs, person‐years; Ref., reference.

* *P* < 0.05.

** *P* < 0.01.

*** *P* < 0.001.

**Table 3:** The association between swallowing problems and cause‐specific mortality (*n* = 44 083)

Cause of death based on ICD‐10 chapters	Swallowing problems	Incidence rate per 1000 PYs	Crude model	ATE‐IPW model^a^
			HR (95% CI)	HR (95% CI)
All causes	No	19.53	1 (Ref.)	1 (Ref.)
	Yes	27.44	1.41 (1.32, 1.51)***	1.14 (1.06, 1.22)***
1. Certain infectious and parasitic diseases	No	0.42	1 (Ref.)	1 (Ref.)
	Yes	0.69	1.65 (1.07, 2.54)*	1.17 (0.73, 1.86)
2. Neoplasms	No	8.33	1 (Ref.)	1 (Ref.)
	Yes	10.46	1.26 (1.13, 1.40)***	1.11 (0.99, 1.24)
4. Endocrine, nutritional and metabolic diseases	No	0.21	1 (Ref.)	1 (Ref.)
	Yes	0.21	0.98 (0.47, 2.05)	0.64 (0.29, 1.41)
5. Mental and behavioral disorders	No	0.09	1 (Ref.)	1 (Ref.)
	Yes	0.19	2.23 (0.98, 5.08)	1.93 (0.81, 4.61)
6. Diseases of the nervous system	No	0.26	1 (Ref.)	1 (Ref.)
	Yes	0.81	3.23 (2.09, 4.99)***	2.84 (1.80, 4.47)***
9. Diseases of the circulatory system	No	4.61	1 (Ref.)	1 (Ref.)
	Yes	5.94	1.30 (1.13, 1.49)***	0.99 (0.85, 1.15)
10. Diseases of the respiratory system	No	2.55	1 (Ref.)	1 (Ref.)
	Yes	4.77	1.89 (1.60, 2.22)***	1.42 (1.20, 1.70)***
11. Diseases of the digestive system	No	0.56	1 (Ref.)	1 (Ref.)
	Yes	0.99	1.78 (1.22, 2.60)**	1.34 (0.92, 1.97)
14. Diseases of the genitourinary system	No	0.35	1 (Ref.)	1 (Ref.)
	Yes	0.54	1.54 (0.94, 2.53)	1.12 (0.66, 1.88)
19. Injury, poisoning and certain other consequences of external causes	No	0.99	1 (Ref.)	1 (Ref.)
	Yes	1.45	1.11 (0.80, 1.54)	0.83 (0.58, 1.19)

^a^ Inverse probability weighting was conducted by using average treatment weight calculated by propensity score of each exposure variable. Each propensity score was estimated by all covariates, including sex, age, equivalent income, education years, number of comorbidities, number of remaining teeth, denture use, smoking status, alcohol consumption, marital status and daily walking time.

Abbreviations: 95% CI, 95% confidence interval; ATE, average treatment effect; HR, hazard ratio; ICD‐10, International Classification of Diseases and Related Health Problems ver. 10; IPW, inverse probability weighted; PYs, person‐years; Ref., reference.

* *P* < 0.05.

** *P* < 0.01.

*** *P* < 0.001.

**Table 4:** Association between xerostomia and cause‐specific mortality (*n* = 44 083)

Cause of death based on ICD‐10 chapters	Xerostomia	Incidence rate per 1000 PYs	Crude model	ATE‐IPW model^a^
			HR (95% CI)	HR (95% CI)
All causes	No	18.94	1 (Ref.)	1 (Ref.)
	Yes	27.78	1.48 (1.39, 1.57)***	1.17 (1.09, 1.25)***
1. Certain infectious and parasitic diseases	No	0.39	1 (Ref.)	1 (Ref.)
	Yes	0.73	1.89 (1.29, 2.77)**	1.42 (0.95, 2.12)
2. Neoplasms	No	8.18	1 (Ref.)	1 (Ref.)
	Yes	10.51	1.29 (1.17, 1.42)***	1.09 (0.98, 1.20)
4. Endocrine, nutritional and metabolic diseases	No	0.20	1 (Ref.)	1 (Ref.)
	Yes	0.27	1.37 (0.75, 2.52)	0.86 (0.46, 1.61)
5. Mental and behavioral disorders	No	0.10	1 (Ref.)	1 (Ref.)
	Yes	0.11	1.09 (0.44, 2.71)	0.80 (0.31, 2.08)
6. Diseases of the nervous system	No	0.27	1 (Ref.)	1 (Ref.)
	Yes	0.60	2.25 (1.44, 3.51)***	1.87 (1.20, 2.92)**
9. Diseases of the circulatory system	No	4.31	1 (Ref.)	1 (Ref.)
	Yes	6.75	1.58 (1.40, 1.79)***	1.20 (1.06, 1.37)**
10. Diseases of the respiratory system	No	2.49	1 (Ref.)	1 (Ref.)
	Yes	4.47	1.81 (1.56, 2.11)***	1.35 (1.16, 1.58)***
11. Diseases of the digestive system	No	0.55	1 (Ref.)	1 (Ref.)
	Yes	0.92	1.68 (1.19, 2.38)**	1.26 (0.88, 1.80)
14. Diseases of the genitourinary system	No	0.34	1 (Ref.)	1 (Ref.)
	Yes	0.54	1.61 (1.04, 2.49)*	1.22 (0.78, 1.91)
19. Injury, poisoning and certain other consequences of external causes	No	0.98	1 (Ref.)	1 (Ref.)
	Yes	1.40	1.30 (0.98, 1.73)	1.08 (0.81, 1.46)

^a^ Inverse probability weighting was conducted by using average treatment weight calculated by propensity score of each exposure variable. Each propensity score was estimated by all covariates, including sex, age, equivalent income, education year, number of comorbidities, number of remaining teeth, denture use, smoking status, alcohol consumption, marital status and daily walking time.

Abbreviations: 95% CI, 95% confidence interval; ATE, average treatment effect; HR, hazard ratio; ICD‐10, International Classification of Diseases and Related Health Problems ver. 10; IPW, inverse probability weighted; PY, person‐years; Ref., reference.

* *P* < 0.05.

** *P* < 0.01.

*** *P* < 0.001.

The results obtained from the additional analysis using data on the causes of death based on a simplified classification system are presented in Supplementary Tables S6–S8. Although we did not observe a significant association between each exposure and the cause of death using a simplified classification system for most causes of death, for the causes classified under the ICD‐10 chapters where significant positive associations were found, the point estimates were positive.

## Discussion

This study evaluated the association between self‐reported poor oral functioning (chewing difficulty, swallowing problems and xerostomia) and mortality risk due to various causes, based on a cohort design. The results suggest that chewing difficulty, swallowing problems and xerostomia were significantly associated with an increased risk of all‐cause mortality. For cause‐specific mortality, chewing difficulty was associated with increased mortality risks due to neoplasms and cardiovascular diseases; swallowing problems were associated with increased mortality risks due to nervous system and respiratory diseases; and xerostomia was associated with increased mortality risks due to nervous system, circulatory and respiratory diseases.

The results of the present study are consistent with these previous findings. Previous studies have reported an association between poor oral functioning and increased risk of all‐cause mortality, such as masticatory dysfunction,^7^ swallowing dysfunction^8^ and hyposalivation.^9^ The present study also observed significant positive associations between poor oral functioning, including chewing difficulty, swallowing problems and xerostomia, and increased all‐cause mortality risk. Additionally, we used various types of cause‐specific mortality as outcome variables. We confirmed that the contribution of the cause of death to all‐cause mortality differed according to the type of oral function.

To interpret the present results, we must note that poor oral functioning did not necessarily cause the disease that led to mortality. Based on the potential outcome framework, this result can be interpreted as poor oral functioning being associated with a higher risk of mortality due to a specific cause.^32^ Therefore, our results suggest that poor oral functioning accelerates the risk of mortality due to specific diseases.

The deterioration of chewing efficiency leads to restricted food intake and malnutrition, including sarcopenia, among older adults.^33^, ^34^ Sarcopenia is associated with poor survival rates and treatment outcomes in various diseases.^35^ Therefore, malnutrition caused by chewing difficulty might increase the risk of mortality from various diseases. In the present study, chewing difficulty was associated with the mortality risk due to neoplasms and cardiovascular diseases. Systematic evidence suggests that malnutrition is a risk factor for poor survival in patients with cancer and coronary artery disease.^36^, ^37^ Therefore, patients with chewing difficulties are at a high risk of death due to these diseases. However, in the present study, the point estimates of all other causes of death also showed a positive association with chewing difficulty. For all other diseases, chewing difficulty could also increase mortality risk by causing malnutrition. Additionally, we found a positive association between xerostomia and an increased risk of death due to cardiovascular disease. Xerostomia also leads to malnutrition,^26^ and malnutrition due to xerostomia would be one of the possible mechanisms between xerostomia and death by cardiovascular diseases.

For swallowing problems, choking while eating or drinking is considered a manifestation of dysphagia.^38^ Our results showed a significant association between swallowing problems and mortality due to respiratory diseases. Dysphagia is a risk factor of aspiration pneumonia.^38^ Hence, individuals with swallowing problems are at an increased risk of mortality owing to respiratory diseases. Dry mouth can also increase the risk of aspiration pneumonia, because reduced salivary secretion increases pneumonia‐associated bacteria in the oral cavity.^39^, ^40^ This study also found that xerostomia is associated with a heightened risk of respiratory diseases. Our findings also suggest that swallowing difficulties and xerostomia are significantly associated with an increased risk of mortality due to nervous system diseases. Aspiration pneumonia is a major direct cause of death in patients with Alzheimer's disease,^41^ and both dysphagia and dry mouth increase the risk of developing aspiration pneumonia. Therefore, it is considered that swallowing difficulties and xerostomia were also significantly associated with the increased risk of mortality due to nervous system diseases as cause of death in the present study.

The present study used the chapter‐based cause‐specific mortality of ICD‐10 as the outcome, and could not investigate more detailed causes of mortality. Future studies with larger sample sizes and causal mediation analyses are required to elucidate the detailed mechanisms underlying the association between poor oral functioning and cause‐specific mortality.

Our results suggest that poor oral function can increase the risk of certain diseases and disorders. Malnutrition and aspiration pneumonia are possible mediating factors in the relationship between poor oral function and increased mortality risk. Oral frailty is “an age‐related phenomenon reflected in decreased oral function^42^” and highlights the importance of poor oral functioning as a manifestation of underlying critical health risks.^43^ To maintain favorable health conditions later in life, dental professionals need to identify those with poor oral functioning and treat them with other medical professionals considering the potential health risks. Additionally, the development of effective interventions to improve oral function among older adults is required.

The present study had several limitations. First, regarding the representativeness of the participants, the distribution of the baseline characteristics might differ from that of the target population because of the moderate response rate. However, we collected data from 11 municipalities by distributing questionnaires to all or to randomly selected eligible residents. Therefore, violations of participants' representativeness were considered small in the present study. Additionally, 98.5% of the participants were connected to death statistics data, leading to less selection bias during the follow‐up period. Second, regarding information bias, we used self‐reported poor oral function as exposure, which might have induced misclassification of the actual condition. Additionally, regarding the cause of death as an outcome variable, a previous study reported that the accuracy of the cause of death in death statistics varies according to the cause of death.^44^ Notably, misclassification of the variables used might have affected the present results. Although misclassifications of exposure and outcome variables existed, these variables were obtained from different data sources, and the degree of misclassification was not considered to be correlated among these variables. Therefore, misclassification was considered non‐differential, and the estimates in the present results would have been biased toward null.^45^ Even under such a bias, we observed significant associations. Stronger associations would be observed in studies with more accurate measures. Although we observed a significant positive association between self‐reported poor oral functioning and cause‐specific mortality, the inaccuracy related to self‐report remains. Future studies using a clinical evaluation of poor oral functioning would strengthen our findings. Third, although we included possible confounders in the statistical analysis, unmeasured confounding factors might have affected the present results. The *E*‐values of the present results show that an unmeasured confounder requires a relatively large association with both the outcome and exposure to explain the observed associations. Therefore, the results are robust against unmeasured confounders.

However, our study had several strengths. Our study enrolled a large number of participants and was followed up for a longer period. Although most previous studies have evaluated the association between poor oral health and death due to cancer, cardiovascular disease or respiratory diseases, which are common causes of death among older adults,^10^, ^11^, ^12^, ^13^, ^14^ we could evaluate various types of associations with cause‐specific mortality, which is due to the large sample size with longer follow‐up data of the present study compared with previous studies.

This exploratory study investigated the association between self‐reported poor oral functioning (chewing difficulty, swallowing problems and xerostomia) and cause‐specific mortality in older adults. The present results showed that chewing difficulty was associated with increased mortality risks due to neoplasms and cardiovascular diseases; swallowing problems were associated with increased mortality risks due to nervous system and respiratory diseases; and xerostomia was associated with increased mortality risks due to nervous system, circulatory and respiratory diseases. Our findings indicated the possibility that poor oral functioning is a potential risk factor of subsequent critical health risks. Dental and medical professionals should pay more attention to patients with poor oral functioning to maintain favorable health conditions later in life.

## Disclosure statement

The authors declare no conflict of interest.

## Author contributions

Taro Kusama, Yudai Tamada, Sakura Kiuchi and Kenji Takeuchi: analysis. Masashige Saito, Toshiyuki Ojima, Jun Aida, Katsunori Kondo and Ken Osaka: acquisition of data. Taro Kusama, Yudai Tamada, Sakura Kiuchi, Masashige Saito, Toshiyuki Ojima, Jun Aida, Katsunori Kondo, Ken Osaka and Kenji Takeuchi: conception, design, interpretation of data, drafting and critical revision of the manuscript. All authors gave their final approval and agreed to be accountable for all aspects of the work.

## Ethics statement

The JAGES survey in 2010 and following survey was approved by the Ethics Committee on Research of Human Subjects at Nihon Fukushi University (10–05), Chiba University Graduate School of Medicine (No. M10460), Tohoku University Graduate School of Dentistry (No. 37582) and Japan Agency for Gerontological Evaluation Study (No. 2025–02).

## Funding

This study was supported by Grant‐in‐Aid for Scientific Research (20H00557, 20K10540, 21H03153, 21H03196, 21K17302, 22H00934, 22H03299, 22K04450, 22K13558, 22K17409, 22K17265, 23K18370, 23K24557, 23H00449, 23H03117) from Japan Society for the Promotion of Science, Health Labor Sciences Research Grants (19FA1012, 19FA2001, 21FA1012, 22FA2001, 22FA1010, 22FG2001, 23FA1022), Research Institute of Science and Technology for Society (JPMJOP1831) from the Japan Science and Technology, a grant from Japan Health Promotion & Fitness Foundation, TMDU priority research areas grant, and National Research Institute for Earth Science and Disaster Resilience. The views and opinions expressed in this article are those of the authors, and do not necessarily reflect the official policy or position of the respective funding organizations.

## Patient consent statement

Informed consent was obtained from all participants.

## Supporting information


**Supplementary Table S1.** Distribution of baseline characteristic by the presence of chewing difficulty before/after inverse probability weighting (*n* = 44 083).
**Supplementary Table S2.** Distribution of baseline characteristic by the presence of swallowing problems before/after inverse probability weighting (*n* = 44 083).
**Supplementary Table S3.** Distribution of baseline characteristic by the presence of xerostomia before/after inverse probability weighting (*n* = 44 083).
**Supplementary Table S4.** Robustness to unmeasured confounding (E‐values) of the association between poor oral functioning and cause‐specific mortality (*n* = 44 083).
**Supplementary Table S5.** The association between poor oral functioning and cause‐specific mortality among complete cases (*n* = 25 986).
**Supplementary Table S6.** The association between chewing difficulty and cause‐specific mortality by a simplified classification system (*n* = 44 083).
**Supplementary Table S7.** The association between swallowing problems and cause‐specific mortality by a simplified classification system (*n* = 44 083).
**Supplementary Table S8.** The association between xerostomia and cause‐specific mortality by a simplified classification system (*n* = 44 083).

## Data Availability

Data were obtained from the JAGES study. All inquiries will be addressed by the Data Management Committee via e‐mail: dataadmin.ml@jages.net. All JAGES datasets have ethical or legal restrictions for public deposition owing to the inclusion of sensitive information from human participants.
