# Supplementary material for: Chewing difficulty, swallowing problems, xerostomia and cause‐specific mortality among older adults: A 7‐year follow‐up JAGES cohort study
Source: Geriatr Gerontol Int. 2025 Aug 25;25(10):1332–40. doi: 10.1111/ggi.70150 (PMC12501720; doi:10.1111/ggi.70150)
Supplement: Supplementary file 1 — Supplementary Table S1. Distribution of baseline characteristic by the presence of chewing difficulty before/after inverse probability weighting (n = 44 083). Supplementary Table S2. Distribution of baseline characteristic by the presence of swallowing problems before/after inverse probability weighting (n = 44 083). Supplementary Table S3. Distribution of baseline characteristic by the presence of xerostomia before/after inverse probability weighting (n = 44 083). Supplementary Table S4. Robustness to unmeasured confounding (E‐values) of the association between poor oral functioning and cause‐specific mortality (n = 44 083). Supplementary Table S5. The association between poor oral functioning and cause‐specific mortality among complete cases (n = 25 986). Supplementary Table S6. The association between chewing difficulty and cause‐specific mortality by a simplified classification system (n = 44 083). Supplementary Table S7. The association between swallowing problems and cause‐specific mortality by a simplified classification system (n = 44 083). Supplementary Table S8. The association between xerostomia and cause‐specific mortality by a simplified classification system (n = 44 083). [file GGI-25-1332-s001.docx]

***Supplementary materials***

**Title: Chewing Difficulty, Swallowing Problems, Xerostomia, and Cause-specific Mortality among Older Adults: A 7-year Follow-up JAGES Cohort Study**

Authors: Taro Kusama, DDS, PhD ^1,2^, Yudai Tamada, PhD ^2,3^, Sakura Kiuchi, DDS, PhD ^2,4^, Masashige, PhD Saito ^5^, Toshiyuki Ojima, MD PhD ^6^, Jun Aida, DDS, PhD ^7^, Katsunori Kondo, MD PhD ^8, 9^, Ken Osaka, MD PhD ^2^, Kenji Takeuchi, DDS, PhD ^1,2^.

Affiliation:

1) Division of Statistics and Data Science, Liaison Center for Innovative Dentistry, Tohoku University Graduate School of Dentistry, Miyagi, Japan

2) Department of International and Community Oral Health, Tohoku University Graduate School of Dentistry, Miyagi, Japan

3) Department of Preventive Medicine, Nagoya University Graduate School of Medicine, Aichi, Japan

4) Frontier Research Institute for Interdisciplinary Sciences, Tohoku University, Miyagi, Japan

5) Faculty of Social Welfare, Nihon Fukushi University, Aichi, Japan

6) Department of Community Health and Preventive Medicine, Hamamatsu University School of Medicine, Shizuoka, Japan.

7) Department of Oral Health Promotion, Graduate School of Medical and Dental Sciences, Tokyo Medical and Dental University, Tokyo, Japan

8) Center for Preventive Medical Sciences, Chiba University, Chiba, Japan.

9) Research Department, Institute for Health Economics and Policy, Tokyo, Japan

*Corresponding author:

Kenji Takeuchi,

Department of International and Community Oral Health

Tohoku University Graduate School of Dentistry

4-1 Seiryo-machi, Aoba-ku, Sendai 980-8575, Japan

Phone: +81-22-717-7639

E-mail: kenji.takeuchi.c4@tohoku.ac.jp

**Supplementary Table S1. Distribution of baseline characteristic by the presence of chewing difficulty before/after inverse probability weighting (n = 44,083)**

| Characteristics |  | Unweighted | | |  | ATE weighted | | |
| --- | --- | --- | --- | --- | --- | --- | --- | --- |
|  |  | Chewing difficulty | | Std. Dif. |  | Chewing difficulty | | Std. Dif. |
|  |  | No | Yes |  |  | No | Yes |  |
|  |  | % | % |  |  | % | % |  |
|  | Total | 100.0 | 100.0 | - |  | 100.0 | 100.0 | - |
| Sex | Male | 46.3 | 48.1 | 0.037 |  | 46.8 | 46.8 | 0.001 |
|  | Female | 53.7 | 51.9 | 0.037 |  | 53.2 | 53.2 | 0.001 |
| Age (year) | 65–69 | 31.8 | 23.9 | 0.177 |  | 29.6 | 29.7 | 0.001 |
|  | 70–74 | 30.8 | 27.5 | 0.073 |  | 29.9 | 29.5 | 0.008 |
|  | 75–79 | 21.5 | 24.6 | 0.075 |  | 22.3 | 22.3 | 0.000 |
|  | 80–84 | 11.3 | 15.8 | 0.133 |  | 12.5 | 12.6 | 0.003 |
|  | ≥85 | 4.7 | 8.2 | 0.142 |  | 5.7 | 5.9 | 0.009 |
| Equivalent income (million JPY) | <2.00 | 47.6 | 57.4 | 0.197 |  | 50.2 | 50.1 | 0.003 |
|  | 2.00–3.99 | 40.6 | 34.2 | 0.133 |  | 38.9 | 39.1 | 0.004 |
|  | ≥4.00 | 11.8 | 8.4 | 0.112 |  | 10.9 | 10.8 | 0.002 |
| Education (year) | ≤9 | 43.3 | 51.9 | 0.173 |  | 45.6 | 45.5 | 0.003 |
|  | 10–12 | 36.8 | 32.8 | 0.085 |  | 35.8 | 36.3 | 0.012 |
|  | ≥13 | 19.9 | 15.3 | 0.120 |  | 18.6 | 18.2 | 0.010 |
| Number of comorbidities | 0 | 26.0 | 22.2 | 0.089 |  | 25.0 | 24.9 | 0.001 |
|  | 1 | 32.3 | 27.0 | 0.116 |  | 30.9 | 30.7 | 0.003 |
|  | 2 | 22.1 | 21.6 | 0.011 |  | 21.9 | 22.0 | 0.001 |
|  | ≥3 | 19.7 | 29.2 | 0.225 |  | 22.2 | 22.4 | 0.005 |
| Number of remaining teeth | ≥20 | 43.1 | 15.0 | 0.651 |  | 35.5 | 34.7 | 0.016 |
|  | ≤19 | 56.9 | 85.0 | 0.651 |  | 64.5 | 65.3 | 0.016 |
| Denture use | No | 50.5 | 33.9 | 0.340 |  | 45.9 | 44.0 | 0.037 |
|  | Yes | 49.5 | 66.1 | 0.340 |  | 54.1 | 56.0 | 0.037 |
| Smoking status | Never | 61.7 | 55.4 | 0.129 |  | 59.9 | 59.3 | 0.013 |
|  | Past | 28.5 | 30.3 | 0.040 |  | 29.0 | 29.3 | 0.007 |
|  | Current | 9.8 | 14.4 | 0.139 |  | 11.1 | 11.4 | 0.009 |
| Alcohol consumption | Never | 59.9 | 61.3 | 0.028 |  | 60.3 | 60.4 | 0.003 |
|  | Past | 3.2 | 4.2 | 0.050 |  | 3.5 | 3.6 | 0.006 |
|  | Current | 36.9 | 34.6 | 0.048 |  | 36.3 | 36.0 | 0.006 |
| Marital status | With a spouse | 73.7 | 69.1 | 0.102 |  | 72.5 | 72.6 | 0.001 |
|  | Without a spouse | 26.3 | 30.9 | 0.102 |  | 27.5 | 27.4 | 0.001 |
| Walking time (minutes/day) | ≥60 | 33.4 | 25.8 | 0.166 |  | 31.3 | 30.9 | 0.010 |
|  | 30–59 | 36.3 | 33.2 | 0.066 |  | 35.4 | 35.6 | 0.004 |
|  | <30 | 30.3 | 41.0 | 0.225 |  | 33.3 | 33.6 | 0.006 |

*Note:* Standardized difference <0.1 indicates no significant distribution difference in the variable between the exposed and the unexposed.

*Abbreviation:* ATE, average treatment effect; Std. Dif., standardized difference.

**Supplementary Table S2. Distribution of baseline characteristic by the presence of swallowing problems before/after inverse probability weighting (n = 44,083)**

| Characteristics |  | Unweighted | | |  | ATE weighted | | |
| --- | --- | --- | --- | --- | --- | --- | --- | --- |
|  |  | Swallowing problems | | Std. Dif. |  | Swallowing problems | | Std. Dif. |
|  |  | No | Yes |  |  | No | Yes |  |
|  |  | % | % |  |  | % | % |  |
|  | Total | 100.0 | 100.0 | - |  | 100.0 | 100.0 | - |
| Sex | Male | 46.8 | 46.7 | 0.002 |  | 46.8 | 46.5 | 0.005 |
|  | Female | 53.2 | 53.3 | 0.002 |  | 53.2 | 53.5 | 0.005 |
| Age (year) | 65–69 | 30.7 | 24.0 | 0.150 |  | 29.6 | 29.7 | 0.000 |
|  | 70–74 | 30.4 | 27.5 | 0.064 |  | 29.9 | 29.9 | 0.000 |
|  | 75–79 | 21.9 | 24.6 | 0.064 |  | 22.3 | 22.2 | 0.003 |
|  | 80–84 | 11.9 | 15.7 | 0.108 |  | 12.5 | 12.6 | 0.001 |
|  | ≥85 | 5.1 | 8.3 | 0.127 |  | 5.6 | 5.7 | 0.003 |
| Equivalent income (million JPY) | <2.00 | 49.6 | 53.9 | 0.087 |  | 50.3 | 50.1 | 0.002 |
|  | 2.00–3.99 | 39.4 | 36.5 | 0.059 |  | 38.9 | 38.9 | 0.000 |
|  | ≥4.00 | 11.1 | 9.6 | 0.049 |  | 10.9 | 11.0 | 0.004 |
| Education (year) | ≤9 | 44.9 | 49.5 | 0.092 |  | 45.6 | 45.5 | 0.004 |
|  | 10–12 | 36.2 | 33.1 | 0.066 |  | 35.7 | 35.9 | 0.003 |
|  | ≥13 | 18.9 | 17.5 | 0.037 |  | 18.7 | 18.7 | 0.001 |
| Number of comorbidities | 0 | 26.2 | 18.4 | 0.190 |  | 25.0 | 24.9 | 0.003 |
|  | 1 | 31.9 | 25.4 | 0.143 |  | 30.9 | 31.0 | 0.003 |
|  | 2 | 21.9 | 21.9 | 0.000 |  | 21.9 | 21.9 | 0.000 |
|  | ≥3 | 20.0 | 34.3 | 0.327 |  | 22.2 | 22.2 | 0.001 |
| Number of remaining teeth | ≥20 | 36.7 | 29.2 | 0.160 |  | 35.5 | 35.6 | 0.001 |
|  | ≤19 | 63.3 | 70.8 | 0.160 |  | 64.5 | 64.4 | 0.001 |
| Denture use | No | 47.1 | 40.2 | 0.139 |  | 46.0 | 46.1 | 0.002 |
|  | Yes | 52.9 | 59.8 | 0.139 |  | 54.0 | 53.9 | 0.002 |
| Smoking status | Never | 60.3 | 58.4 | 0.038 |  | 60.0 | 60.1 | 0.002 |
|  | Past | 28.5 | 31.4 | 0.063 |  | 29.0 | 28.9 | 0.002 |
|  | Current | 11.2 | 10.2 | 0.032 |  | 11.0 | 11.0 | 0.002 |
| Alcohol consumption | Never | 60.0 | 61.7 | 0.034 |  | 60.3 | 60.2 | 0.002 |
|  | Past | 3.3 | 4.6 | 0.068 |  | 3.5 | 3.4 | 0.002 |
|  | Current | 36.7 | 33.8 | 0.062 |  | 36.3 | 36.4 | 0.003 |
| Marital status | With a spouse | 73.1 | 69.3 | 0.084 |  | 72.5 | 72.5 | 0.000 |
|  | Without a spouse | 26.9 | 30.7 | 0.084 |  | 27.5 | 27.5 | 0.000 |
| Walking time (minutes/day) | ≥60 | 32.4 | 25.9 | 0.142 |  | 31.3 | 31.4 | 0.002 |
|  | 30–59 | 35.7 | 34.0 | 0.037 |  | 35.5 | 35.4 | 0.001 |
|  | <30 | 31.9 | 40.1 | 0.171 |  | 33.2 | 33.2 | 0.001 |

*Note:* Standardized difference <0.1 indicates no significant distribution difference in the variable between the exposed and the unexposed.

*Abbreviation:* ATE, average treatment effect; Std. Dif., standardized difference.

**Supplementary Table S3. Distribution of baseline characteristic by the presence of xerostomia before/after inverse probability weighting (n = 44,083)**

| Characteristics |  | Unweighted | | |  | ATE weighted | | |
| --- | --- | --- | --- | --- | --- | --- | --- | --- |
|  |  | Xerostomia | | Std. Dif. |  | Xerostomia | | Std. Dif. |
|  |  | No | Yes |  |  | No | Yes |  |
|  |  | % | % |  |  | % | % |  |
|  | Total | 100.0 | 100.0 | - |  | 100.0 | 100.0 | - |
| Sex | Male | 46.9 | 46.4 | 0.009 |  | 46.8 | 46.8 | 0.001 |
|  | Female | 53.1 | 53.6 | 0.009 |  | 53.2 | 53.3 | 0.001 |
| Age (year) | 65–69 | 31.2 | 24.0 | 0.161 |  | 29.7 | 29.7 | 0.000 |
|  | 70–74 | 30.4 | 27.9 | 0.056 |  | 29.9 | 29.6 | 0.006 |
|  | 75–79 | 21.6 | 25.2 | 0.085 |  | 22.3 | 22.3 | 0.001 |
|  | 80–84 | 11.7 | 15.5 | 0.110 |  | 12.5 | 12.7 | 0.006 |
|  | ≥85 | 5.1 | 7.5 | 0.096 |  | 5.6 | 5.7 | 0.003 |
| Equivalent income (million JPY) | <2.00 | 48.9 | 55.4 | 0.132 |  | 50.2 | 50.0 | 0.005 |
|  | 2.00–3.99 | 39.9 | 35.2 | 0.097 |  | 38.9 | 39.1 | 0.003 |
|  | ≥4.00 | 11.3 | 9.4 | 0.062 |  | 10.9 | 11.0 | 0.003 |
| Education (year) | ≤9 | 44.4 | 50.2 | 0.116 |  | 45.6 | 45.5 | 0.002 |
|  | 10–12 | 36.4 | 33.3 | 0.065 |  | 35.7 | 35.6 | 0.003 |
|  | ≥13 | 19.2 | 16.5 | 0.071 |  | 18.7 | 18.9 | 0.006 |
| Number of comorbidities | 0 | 27.0 | 17.4 | 0.234 |  | 25.0 | 24.8 | 0.004 |
|  | 1 | 32.5 | 24.8 | 0.169 |  | 30.9 | 31.0 | 0.002 |
|  | 2 | 21.8 | 22.5 | 0.017 |  | 21.9 | 22.1 | 0.004 |
|  | ≥3 | 18.8 | 35.3 | 0.380 |  | 22.2 | 22.1 | 0.001 |
| Number of remaining teeth | ≥20 | 37.2 | 29.1 | 0.174 |  | 35.5 | 35.4 | 0.001 |
|  | ≤19 | 62.8 | 71.0 | 0.174 |  | 64.5 | 64.6 | 0.001 |
| Denture use | No | 47.2 | 41.5 | 0.115 |  | 46.0 | 45.8 | 0.005 |
|  | Yes | 52.8 | 58.5 | 0.115 |  | 54.0 | 54.2 | 0.005 |
| Smoking status | Never | 60.4 | 58.5 | 0.038 |  | 60.0 | 59.9 | 0.003 |
|  | Past | 29.0 | 29.0 | 0.002 |  | 29.0 | 29.0 | 0.000 |
|  | Current | 10.7 | 12.5 | 0.056 |  | 11.0 | 11.2 | 0.004 |
| Alcohol consumption | Never | 59.8 | 62.2 | 0.049 |  | 60.3 | 60.3 | 0.001 |
|  | Past | 3.2 | 4.4 | 0.059 |  | 3.5 | 3.5 | 0.001 |
|  | Current | 37.0 | 33.5 | 0.074 |  | 36.3 | 36.3 | 0.000 |
| Marital status | With a spouse | 73.5 | 68.8 | 0.104 |  | 72.5 | 72.5 | 0.000 |
|  | Without a spouse | 26.5 | 31.2 | 0.104 |  | 27.5 | 27.5 | 0.000 |
| Walking time (minutes/day) | ≥60 | 32.8 | 25.8 | 0.154 |  | 31.4 | 31.4 | 0.001 |
|  | 30–59 | 36.0 | 33.2 | 0.059 |  | 35.4 | 35.5 | 0.001 |
|  | <30 | 31.2 | 41.0 | 0.205 |  | 33.2 | 33.1 | 0.002 |

*Note:* Standardized difference <0.1 indicates no significant distribution difference in the variable between the exposed and the unexposed.

*Abbreviation:* ATE, average treatment effect; Std. Dif., standardized difference.

**Supplementary Table S4. Robustness to unmeasured confounding (E-values) of the association between poor oral functioning and cause-specific mortality (n = 44,083).**

|  | Chewing difficulty | |  | Swallowing problems | |  | Xerostomia | |
| --- | --- | --- | --- | --- | --- | --- | --- | --- |
| Cause-specific death based on ICD-10 chapter | E-value for point estimate ^a, b^ | E-value for confidence limit ^a, c^ |  | E-value for point estimate ^a, b^ | E-value for confidence limit ^a, c^ |  | E-value for point estimate ^a, b^ | E-value for confidence limit ^a, c^ |
| All causes | 1.62 | 1.43 |  | 1.54 | 1.31 |  | 1.62 | 1.41 |
| 1. Certain infectious  and parasitic diseases | 1.90 | 1.00 |  | 1.62 | 1.00 |  | 2.19 | 1.00 |
| 2. Neoplasms | 1.64 | 1.34 |  | 1.46 | 1.00 |  | 1.40 | 1.00 |
| 4. Endocrine, nutritional  and metabolic diseases | 1.54 | 1.00 |  | 2.50 | 1.00 |  | 1.60 | 1.00 |
| 5. Mental and behavioral disorders | 1.92 | 1.00 |  | 3.27 | 1.00 |  | 1.81 | 1.00 |
| 6. Diseases of the nervous system | 2.47 | 1.00 |  | 5.13 | 3.00 |  | 3.15 | 1.69 |
| 9. Diseases of the circulatory system | 1.57 | 1.11 |  | 1.11 | 1.00 |  | 1.69 | 1.31 |
| 10. Diseases of the respiratory system | 1.24 | 1.00 |  | 2.19 | 1.69 |  | 2.04 | 1.59 |
| 11. Diseases of the digestive system | 1.67 | 1.00 |  | 2.01 | 1.00 |  | 1.83 | 1.00 |
| 14. Diseases of the genitourinary system | 2.04 | 1.00 |  | 1.49 | 1.00 |  | 1.74 | 1.00 |
| 19. Injury, poisoning and certain other  consequences of external causes | 1.64 | 1.00 |  | 1.70 | 1.00 |  | 1.37 | 1.00 |

^a^ Inverse probability weighting was conducted by using average treatment weight calculated by propensity score of each exposure variable. Each propensity score was estimated by all covariates, including sex, age, equivalent income, education year, number of comorbidities, number of remaining teeth, denture use, smoking status, alcohol consumption, marital status, and daily walking time.

^b^ E-values present the minimum strength of association on the risk ratio scale that an unmeasured confounder would need to have with both exposure and outcome to fully explain away the observed association conditional on included covariates.

^c^ E-values for the 95% confident interval limit closest to the null denotes the minimum strength of association on the risk ratio scale that an unmeasured confounder would need to have with both exposure and the outcome to shift the 95% confident interval to include the null value conditional on included covariates.

Abbreviations: ICD-10, International Classification of Diseases and Related Health Problems ver. 10.

**Supplementary Table S5. The association between poor oral functioning and cause-specific mortality among complete cases (n = 25,986)**

| Cause-specific death based on ICD-10 chapter | Chewing difficulty |  | Swallowing problems |  | Xerostomia |
| --- | --- | --- | --- | --- | --- |
|  | HR (95% CI) ^a^ |  | HR (95% CI) ^a^ |  | HR (95% CI) ^a^ |
|  | 1 (Ref.) |  | 1 (Ref.) |  | 1 (Ref.) |
| All causes | 1.10 (1.01, 1.20)* |  | 1.19 (1.08, 1.31)*** |  | 1.14 (1.04, 1.24)** |
| 1. Certain infectious  and parasitic diseases | 1.04 (0.61, 1.78) |  | 1.17 (0.64, 2.15) |  | 1.23 (0.70, 2.17) |
| 2. Neoplasms | 1.13 (0.99, 1.29) |  | 1.13 (0.98, 1.31) |  | 1.04 (0.91, 1.19) |
| 4. Endocrine, nutritional  and metabolic diseases | 0.88 (0.39, 1.98) |  | 0.82 (0.33, 2.07) |  | 0.62 (0.25, 1.53) |
| 5. Mental and behavioral disorders | 0.85 (0.23, 3.15) |  | 1.11 (0.30, 4.14) |  | 0.46 (0.10, 2.19) |
| 6. Diseases of the nervous system | 1.63 (0.89, 3.01) |  | 4.15 (2.36, 7.29)*** |  | 1.68 (0.93, 3.02) |
| 9. Diseases of the circulatory system | 1.09 (0.91, 1.29) |  | 0.95 (0.77, 1.16) |  | 1.20 (1.00, 1.43)* |
| 10. Diseases of the respiratory system | 1.03 (0.83, 1.27) |  | 1.64 (1.31, 2.04)*** |  | 1.39 (1.12, 1.72)** |
| 11. Diseases of the digestive system | 1.11 (0.69, 1.77) |  | 1.84 (1.14, 2.98)** |  | 0.99 (0.59, 1.66) |
| 14. Diseases of the genitourinary system | 1.09 (0.62, 1.93) |  | 1.24 (0.67, 2.30) |  | 1.41 (0.79, 2.55) |
| 19. Injury, poisoning and certain other  consequences of external causes | 0.94 (0.65, 1.36) |  | 0.74 (0.46, 1.18) |  | 0.99 (0.65, 1.49) |

^a^ Inverse probability weighting was conducted by using average treatment weight calculated by propensity score of each exposure variable. Each propensity score was estimated by all covariates, including sex, age, equivalent income, education year, number of comorbidities, number of remaining teeth, denture use, smoking status, alcohol consumption, marital status, and daily walking time.

Abbreviations: ICD-10, International Classification of Diseases and Related Health Problems ver. 10; HR, hazard ratio; 95% CI, 95% confidence interval; Ref., reference.

*p<0.05, **p<0.01, ***p<0.001

**Supplementary Table S6. The association between chewing difficulty and cause-specific mortality by a simplified classification system (n = 44,083)**

| Causes of death based on a simplified classification system | Incidence rate (per 10,000 person-year) | | PS adjusted model ^a^ | | | |
| --- | --- | --- | --- | --- | --- | --- |
|  | Chewing difficulty | | HR ^b^ | 95% CI | | Sig. |
|  | No | Yes |  | LL | UL |  |
| Tracheobronchial & lung cancer | 16.84 | 25.16 | 1.02 | 0.84 | 1.24 | NS |
| Pneumonia | 12.19 | 19.46 | 0.99 | 0.80 | 1.23 | NS |
| Stomach cancer | 9.74 | 14.17 | 1.24 | 0.96 | 1.59 | NS |
| Heart failure | 6.70 | 14.71 | 1.44 | 1.10 | 1.89 | * |
| Pancreatic cancer | 8.41 | 10.91 | 1.13 | 0.84 | 1.52 | NS |
| Colon cancer | 6.42 | 8.28 | 1.05 | 0.76 | 1.44 | NS |
| Cerebral infarction | 5.25 | 9.23 | 1.18 | 0.86 | 1.62 | NS |
| Liver cancer | 5.65 | 7.84 | 1.03 | 0.73 | 1.46 | NS |
| Senility | 4.33 | 9.45 | 1.37 | 0.97 | 1.92 | NS |
| Acute myocardial infarction | 5.53 | 6.41 | 0.79 | 0.54 | 1.16 | NS |
| Arrhythmia | 4.67 | 7.84 | 1.22 | 0.86 | 1.72 | NS |
| Intracerebral hemorrhage | 4.16 | 6.00 | 1.10 | 0.75 | 1.62 | NS |
| Gallbladder cancer | 3.90 | 4.67 | 0.95 | 0.62 | 1.47 | NS |
| Aortic aneurysm | 2.89 | 4.61 | 1.11 | 0.71 | 1.74 | NS |
| Malignant lymphoma | 2.68 | 6.43 | 1.78 | 1.17 | 2.70 | * |
| Chronic obstructive pulmonary disease | 2.55 | 5.16 | 1.20 | 0.77 | 1.88 | NS |
| Rectal cancer | 2.40 | 3.12 | 1.07 | 0.64 | 1.81 | NS |
| Esophageal cancer | 2.62 | 3.07 | 0.91 | 0.53 | 1.54 | NS |
| Subarachnoid hemorrhage | 2.08 | 1.98 | 0.78 | 0.37 | 1.66 | NS |
| Leukemia | 1.85 | 3.28 | 1.57 | 0.89 | 2.77 | NS |
| Sepsis | 1.54 | 2.80 | 1.46 | 0.80 | 2.65 | NS |
| Chronic kidney disease | 1.45 | 2.77 | 1.27 | 0.67 | 2.42 | NS |
| Fall | 1.60 | 2.63 | 1.29 | 0.71 | 2.34 | NS |
| Suicide | 1.50 | 2.90 | 1.36 | 0.76 | 2.43 | NS |
| Oropharyngeal cancer | 1.36 | 2.88 | 1.42 | 0.74 | 2.75 | NS |
| Bladder cancer | 1.53 | 2.14 | 1.01 | 0.54 | 1.90 | NS |
| Chronic non-rheumatic valvular heart disease | 1.18 | 2.31 | 1.29 | 0.66 | 2.51 | NS |
| Drowning | 1.44 | 2.12 | 1.25 | 0.64 | 2.46 | NS |
| Diabetes | 1.11 | 1.41 | 0.80 | 0.36 | 1.79 | NS |
| Asphyxia | 1.22 | 2.06 | 1.10 | 0.54 | 2.23 | NS |
| Traffic accident | 0.79 | 1.87 | 2.04 | 0.94 | 4.43 | NS |
| Vascular dementia | 0.87 | 1.25 | 0.91 | 0.39 | 2.12 | NS |
| Alzheimer's disease | 0.77 | 1.25 | 0.90 | 0.37 | 2.21 | NS |
| Cardiomyopathy | 0.62 | 1.41 | 1.33 | 0.55 | 3.20 | NS |

^a^ Adjusted for propensity score estimated by all covariates, including sex, age, equivalent income, education year, number of comorbidities, number of remaining teeth, denture use, smoking status, alcohol consumption, marital status, and daily walking time.

^b^ Ref. HR = 1

Abbreviations: PS, propensity score; HR, hazard ratio; 95% CI, 95% confidence interval; LL, lower limit; UL, upper limit; Ref., reference; Sig., significance; NS, not significant.

*p<0.05, **p<0.01, ***p<0.001

**Supplementary Table S7. The association between swallowing problems and cause-specific mortality by a simplified classification system (n = 44,083)**

| Causes of death based on a simplified classification system | Incidence rate (per 10,000 person-year) | | PS adjusted model ^a^ | | | |
| --- | --- | --- | --- | --- | --- | --- |
|  | Swallowing problems | | HR ^b^ | 95% CI | | Sig. |
|  | No | Yes |  | LL | UL |  |
| Tracheobronchial & lung cancer | 18.19 | 23.72 | 1.07 | 0.84 | 1.36 | NS |
| Pneumonia | 12.61 | 22.24 | 1.24 | 0.97 | 1.59 | NS |
| Stomach cancer | 10.88 | 11.08 | 0.90 | 0.64 | 1.26 | NS |
| Heart failure | 7.86 | 14.03 | 1.25 | 0.92 | 1.71 | NS |
| Pancreatic cancer | 8.74 | 10.89 | 1.15 | 0.82 | 1.61 | NS |
| Colon cancer | 6.72 | 7.98 | 1.01 | 0.68 | 1.49 | NS |
| Cerebral infarction | 5.81 | 8.95 | 1.13 | 0.77 | 1.64 | NS |
| Liver cancer | 5.70 | 9.09 | 1.19 | 0.82 | 1.71 | NS |
| Senility | 4.98 | 9.51 | 1.30 | 0.88 | 1.93 | NS |
| Acute myocardial infarction | 5.45 | 7.48 | 1.07 | 0.72 | 1.60 | NS |
| Arrhythmia | 5.39 | 6.14 | 0.90 | 0.58 | 1.39 | NS |
| Intracerebral hemorrhage | 4.34 | 6.28 | 1.20 | 0.78 | 1.86 | NS |
| Gallbladder cancer | 3.99 | 4.71 | 1.06 | 0.62 | 1.81 | NS |
| Aortic aneurysm | 3.35 | 3.32 | 0.73 | 0.41 | 1.31 | NS |
| Malignant lymphoma | 3.50 | 4.61 | 0.99 | 0.60 | 1.63 | NS |
| Chronic obstructive pulmonary disease | 2.95 | 4.80 | 1.17 | 0.69 | 1.97 | NS |
| Rectal cancer | 2.43 | 3.46 | 1.26 | 0.69 | 2.31 | NS |
| Esophageal cancer | 2.59 | 3.55 | 1.25 | 0.68 | 2.29 | NS |
| Subarachnoid hemorrhage | 2.23 | 1.11 | 0.45 | 0.16 | 1.30 | NS |
| Leukemia | 1.99 | 3.55 | 1.73 | 0.95 | 3.14 | NS |
| Sepsis | 1.78 | 2.40 | 0.96 | 0.48 | 1.94 | NS |
| Chronic kidney disease | 1.55 | 3.14 | 1.44 | 0.71 | 2.95 | NS |
| Fall | 1.90 | 1.71 | 0.71 | 0.27 | 1.85 | NS |
| Suicide | 1.83 | 2.08 | 0.86 | 0.41 | 1.78 | NS |
| Oropharyngeal cancer | 1.47 | 3.37 | 1.87 | 0.95 | 3.67 | NS |
| Bladder cancer | 1.67 | 1.80 | 0.84 | 0.37 | 1.95 | NS |
| Chronic non-rheumatic valvular heart disease | 1.49 | 1.43 | 0.65 | 0.25 | 1.65 | NS |
| Drowning | 1.60 | 1.71 | 0.93 | 0.39 | 2.19 | NS |
| Diabetes | 1.25 | 0.88 | 0.48 | 0.15 | 1.48 | NS |
| Asphyxia | 1.21 | 2.68 | 1.62 | 0.79 | 3.34 | NS |
| Traffic accident | 1.21 | 0.37 | 0.25 | 0.04 | 1.69 | NS |
| Vascular dementia | 0.80 | 1.89 | 1.72 | 0.72 | 4.07 | NS |
| Alzheimer's disease | 0.77 | 1.62 | 1.44 | 0.59 | 3.51 | NS |
| Cardiomyopathy | 0.81 | 0.92 | 0.83 | 0.28 | 2.49 | NS |

^a^ Adjusted for propensity score estimated by all covariates, including sex, age, equivalent income, education year, number of comorbidities, number of remaining teeth, denture use, smoking status, alcohol consumption, marital status, and daily walking time.

^b^ Ref. HR = 1

Abbreviations: PS, propensity score; HR, hazard ratio; 95% CI, 95% confidence interval; LL, lower limit; UL, upper limit; Ref., reference; Sig., significance; NS, not significant.

*p<0.05, **p<0.01, ***p<0.001

**Supplementary Table S8. The association between xerostomia and cause-specific mortality by a simplified classification system (n = 44,083)**

| Causes of death based on a simplified classification system | Incidence rate (per 10,000 person-year) | | PS adjusted model ^a^ | | | |
| --- | --- | --- | --- | --- | --- | --- |
|  | Xerostomia | | HR ^b^ | 95% CI | | Sig. |
|  | No | Yes |  | LL | UL |  |
| Tracheobronchial & lung cancer | 17.45 | 25.20 | 1.15 | 0.92 | 1.43 | NS |
| Pneumonia | 11.99 | 22.30 | 1.34 | 1.08 | 1.68 | * |
| Stomach cancer | 10.24 | 13.49 | 1.19 | 0.91 | 1.55 | NS |
| Heart failure | 7.19 | 15.10 | 1.50 | 1.14 | 1.97 | * |
| Pancreatic cancer | 8.65 | 10.70 | 1.14 | 0.84 | 1.53 | NS |
| Colon cancer | 6.84 | 7.20 | 0.90 | 0.62 | 1.30 | NS |
| Cerebral infarction | 5.60 | 9.02 | 1.18 | 0.84 | 1.65 | NS |
| Liver cancer | 6.10 | 6.75 | 0.77 | 0.52 | 1.13 | NS |
| Senility | 5.13 | 7.83 | 1.10 | 0.76 | 1.58 | NS |
| Acute myocardial infarction | 5.14 | 8.14 | 1.24 | 0.86 | 1.78 | NS |
| Arrhythmia | 5.26 | 6.47 | 0.97 | 0.66 | 1.42 | NS |
| Intracerebral hemorrhage | 4.18 | 6.43 | 1.26 | 0.84 | 1.89 | NS |
| Gallbladder cancer | 4.10 | 4.12 | 0.93 | 0.57 | 1.51 | NS |
| Aortic aneurysm | 2.98 | 4.79 | 1.22 | 0.72 | 2.08 | NS |
| Malignant lymphoma | 3.10 | 5.87 | 1.46 | 0.95 | 2.25 | NS |
| Chronic obstructive pulmonary disease | 2.77 | 5.07 | 1.34 | 0.84 | 2.15 | NS |
| Rectal cancer | 2.42 | 3.25 | 1.20 | 0.68 | 2.12 | NS |
| Esophageal cancer | 2.69 | 2.90 | 0.93 | 0.51 | 1.69 | NS |
| Subarachnoid hemorrhage | 1.94 | 2.48 | 1.19 | 0.52 | 2.74 | NS |
| Leukemia | 2.18 | 2.45 | 1.07 | 0.55 | 2.07 | NS |
| Sepsis | 1.69 | 2.59 | 1.10 | 0.58 | 2.11 | NS |
| Chronic kidney disease | 1.52 | 2.90 | 1.29 | 0.69 | 2.41 | NS |
| Fall | 1.91 | 1.71 | 0.71 | 0.34 | 1.50 | NS |
| Suicide | 1.81 | 2.13 | 0.84 | 0.43 | 1.64 | NS |
| Oropharyngeal cancer | 1.39 | 3.22 | 1.88 | 1.02 | 3.46 | * |
| Bladder cancer | 1.69 | 1.71 | 0.77 | 0.35 | 1.68 | NS |
| Chronic non-rheumatic valvular heart disease | 1.45 | 1.57 | 0.74 | 0.33 | 1.66 | NS |
| Drowning | 1.44 | 2.31 | 1.43 | 0.73 | 2.79 | NS |
| Diabetes | 1.08 | 1.61 | 0.97 | 0.43 | 2.16 | NS |
| Asphyxia | 1.51 | 1.19 | 0.57 | 0.23 | 1.42 | NS |
| Traffic accident | 0.87 | 1.89 | 1.94 | 0.84 | 4.49 | NS |
| Vascular dementia | 0.94 | 1.08 | 0.84 | 0.33 | 2.19 | NS |
| Alzheimer's disease | 0.67 | 1.78 | 1.81 | 0.77 | 4.22 | NS |
| Cardiomyopathy | 0.74 | 1.15 | 1.11 | 0.42 | 2.91 | NS |

^a^ Adjusted for propensity score estimated by all covariates, including sex, age, equivalent income, education year, number of comorbidities, number of remaining teeth, denture use, smoking status, alcohol consumption, marital status, and daily walking time.

^b^ Ref. HR = 1

Abbreviations: PS, propensity score; HR, hazard ratio; 95% CI, 95% confidence interval; LL, lower limit; UL, upper limit; Ref., reference; Sig., significance; NS, not significant.

*p<0.05, **p<0.01, ***p<0.001
